# Supplementary material for: Vasopressin Receptor Type-2 Mediated Signaling in Renal Cell Carcinoma Stimulates Stromal Fibroblast Activation
Source: Int J Mol Sci. 2022 Jul 9;23(14):7601. doi: 10.3390/ijms23147601 (PMC9325308; doi:10.3390/ijms23147601)
Supplement: Supplementary file 1 [file ijms-23-07601-s001.zip › ijms-1784282-supplementary.pdf]

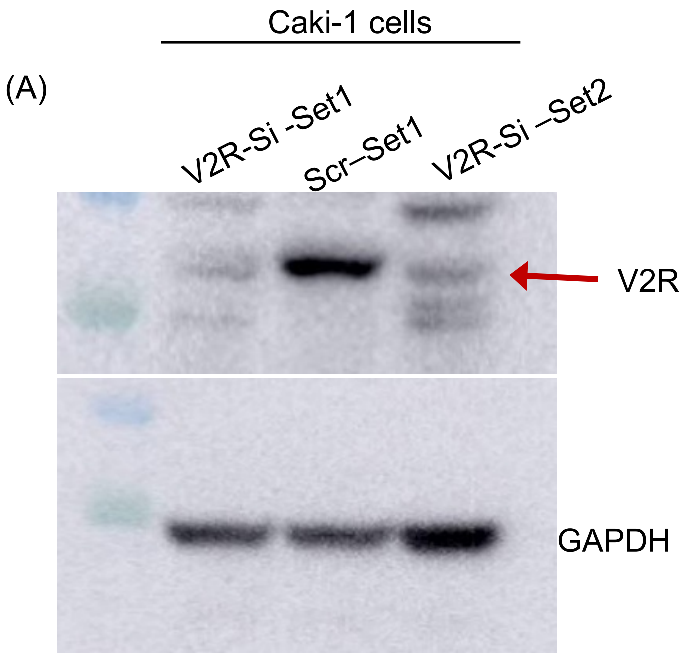

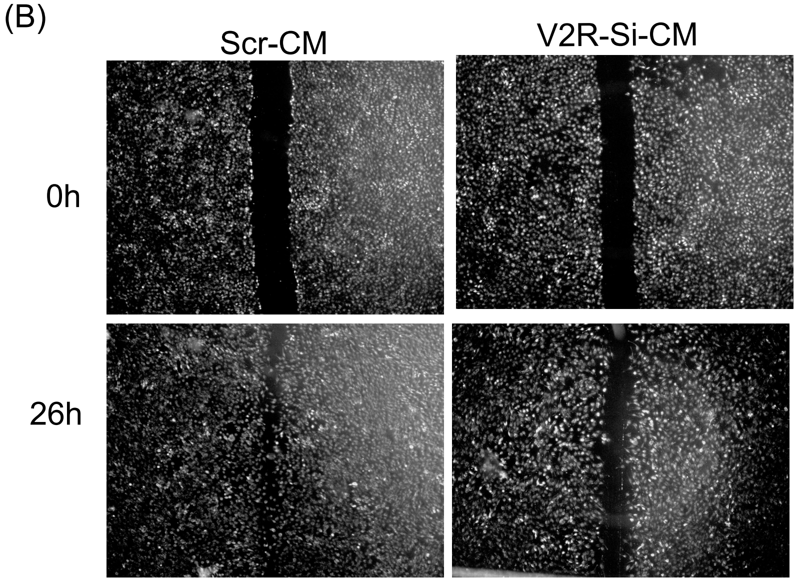

Supplemental Figure S1: (A) Immunoblot shows Caki-1 cells gene silenced for V2R (SiRNA) or Scr (Scrambled RNA). (B) Representative images of wound closure in scratch assay on NRK-49F cells exposed to CM from Scr or V2R-SiRNA transfected Caki-1 cells

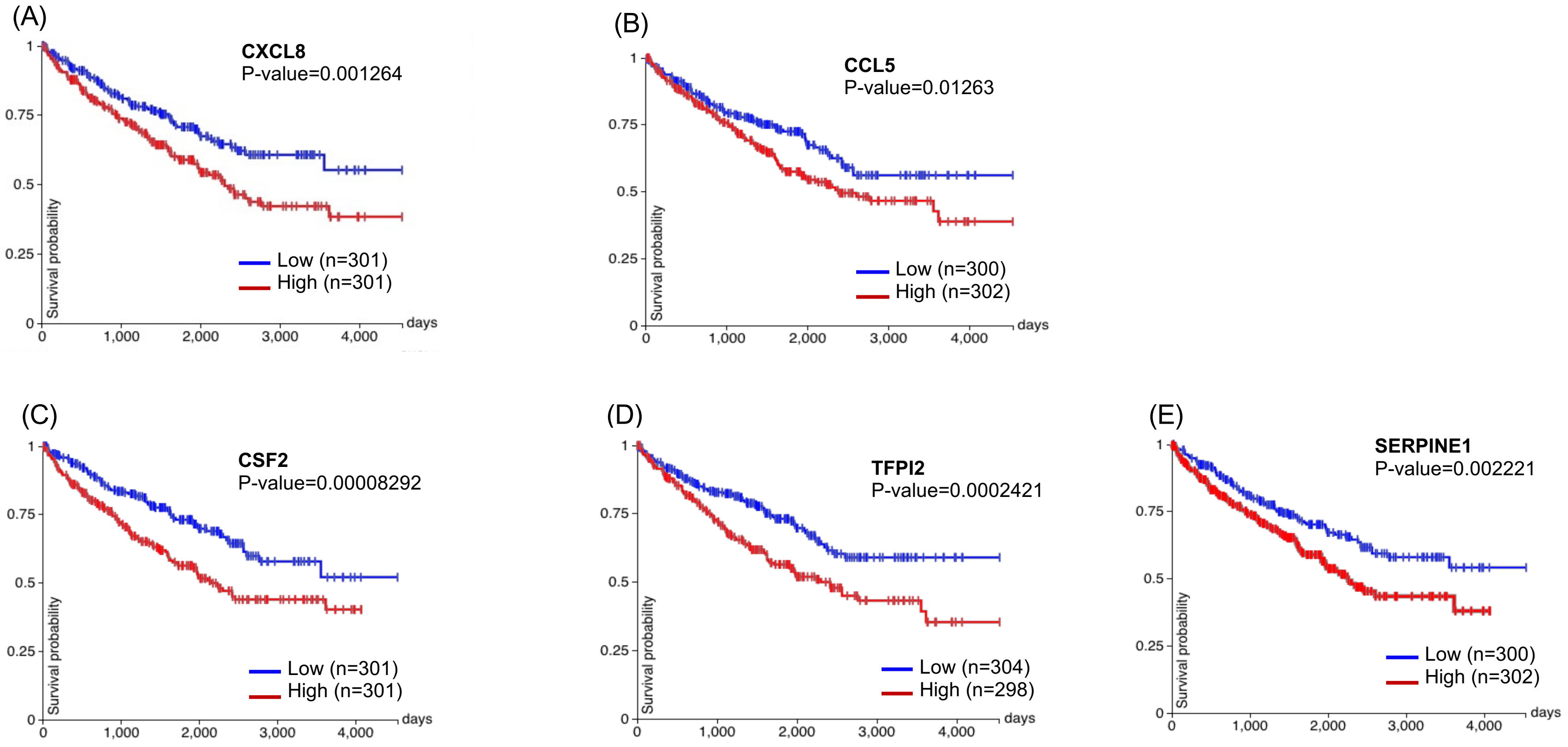

Supplemental Figure S2: Analysis of The Cancer Genome Atlas (TCGA) kidney clear cell carcinoma (KIRC) database for RNA sequencing data related to ccRCC shows significantly reduced overall survival in high expressers of CXCL8 (IL8), CCL5, CSF2, TFPI-2 and SERPINE1 (PAI1).

Supplemental- Table S1:

Human Primers used for QRTPCR:

**Gene Name**	**Primers Forward (F) and Reverse (R)**
CTGF	F CGACTGGAAGACACGT TTGG<br>R AGGCTTGGAGATTTTGGGAG
SPARC	F ATCTAAATCCACTCCTTCCACAG<br>R CACCGTTAATGTATTCACTTAAATC
OPN	F TGAGAGCAATGAGCATTCCGATG<br>R CAGGGAGTTTCCATGAAGCCAC
MMP1	F ACAGCCCAGTACTTATTCCCTTTG<br>R GGGCTTGAAGCTGCTTACGA
PAI-1	F GGCCATTACTACGACATCCTG<br>R GGTCATGTTGCCTTTCCAGT
AREG	F GTGGTGCTGTCGCTCTTGATA<br>R ACTCACAGGGGAAATCTCACT
CYR61	F GAGTGGGTCTGTGACGAGGAT<br>R GGTTGTATAGGATGCGAGGCT
ICAM1	F CCTTCCTCACCGTGTACTGG<br>R AGCGTAGGGTAAGGTTCTTGC
TSP1	F AACAACCCCACACCCCAGTTTG<br>R TTGAAGCAGGCATCAGTCAC
TIMP1	F GACGGCCTTCTGCAATTCC<br>R GTATAAGGTGGTCTGGTTGACTTCTG
TIMP2	F GAGCCTGAACCACAGGTACCA<br>R TCTGTGACCCAGTCCATCCA
Adamts2	F CTGGCAAGCATTGTTTTAAAGGA<br>R GGAGCCAAACGGACTCCAA
TGF-β	F GAG CCT GAG GCC GAC TAC TA<br>R GGG TTC AGG TAC CGC TTC TC
CSF-1	F CCAGGAACAGTTGAAAGATCCA<br>R TTATCTCTGAAGCCATGGTGT
CSF-2	F CACTGCTGCTGAGATGAATGAAA<br>R GTCTGTAGGCAGGTCGGCTC
PDGF-A	F CCCCTGCCCATTCGGAGGAAGAG<br>R TTGGCCACCTTGACGCTGCGGTG
Cxcl10	F GTGGCATTCAAGGAGTACCTC<br>R TGATGGCCTTCGATTCTGGATT
CCL2	F CCGAGAGGCTGAGACTAAC<br>R CTTGCTGCTGGTGATTCTTC
CCL5	F CCTCGCTGTCATCCTCATTG<br>R GGGTTGGCACACACTTGG

CCL20	F - AAGTTGTCTGTGTGCGCAAATCC<br>R - CCATTCCAGAAAAGCCACAGTTTT
IL1b	F AAACAGATGAAGTGCTCCTTCCAGG<br>R TGGAGAACACCACTTGTTGCTCCA
IL6	F AATTCGGTACATCCTCGACGG<br>R GGTTGTTTTCTGCCAGTGCC
IL8	F GACCACACTGCGCCAACAC<br>R CTTCTCCACAACCCTCTGCAC
TNFa	F CCG AGG CAG TCA GAT CAT CTT<br>R AGC TGC CCC TCA GCT TGA
SEMA7A	F TGTGTATTCCCTCGGTGACA<br>R GAGTGGAACAATGGCGTCTT
TFPI-2	F CCAGATGAAGCTACTTGTATG<br>R GCACATGCACGTTTGCAATC
GAPDH	F CCA GGT GGT CTC CTC TGA CT<br>R TGC TGT AGC CAA ATT CGT TG
β-actin	F CACCATTGGCAATGAGCGGTTC<br>R AGGTCTTTGCGGATGTCCACGT
